# Severe Cerebral Vasospasm Caused by Acute on Top of Chronic Spontaneous Subdural Hematoma: A Case Report and Review of the Literature

**DOI:** 10.7759/cureus.38004

**Published:** 2023-04-23

**Authors:** Badr E Hafiz, Bassem Y Sheikh, Abdulmajeed S Alautabi, Ahmed A Najjar

**Affiliations:** 1 Medicine, Taibah University, Medina, SAU; 2 Neurosurgery, College of Medicine, Taibah University, Medina, SAU; 3 Neurosurgery, King Salman Bin Abdulaziz Medical City, Medina, SAU

**Keywords:** spontaneous subdural hematoma, case report, cerebral ischemia, arterial vasospasm, subarachnoid hemorrhage, subdural hematoma

## Abstract

Cerebral vasospasm is a well-known phenomenon that has been associated with subarachnoid hemorrhage due to aneurysmal bleeding. It can lead to serious outcomes if not recognized and treated promptly. It happens most frequently following cases of aneurysmal subarachnoid hemorrhage. Other causes include traumatic brain injury, reversible cerebral vasoconstriction syndrome, post-tumor resection, and non-aneurysmal subarachnoid hemorrhage. We describe a case of severe clinical vasospasm following acute on top of chronic spontaneous subdural hematoma in a patient with corpus callosum agenesis. Also, a small literature review of the possible risk factors of such occurrence is discussed.

## Introduction

Cerebral vasospasm is a well-known phenomenon in the context of subarachnoid hemorrhage (SAH) secondary to cerebral arterial aneurysms. It is also associated with other pathologies such as traumatic brain injury, reversible cerebral vasoconstriction syndrome, post-tumor resection surgery, and other non-aneurysmal SAHs [1,2]. The occurrence of severe symptomatic clinical vasospasm is infrequent clinically in contexts other than aneurysmal SAH or other forms of severe brain insult [2]. To our knowledge, this case report is the first to report such a complication following a non-traumatic acute on top of chronic non-traumatic subdural hematoma (SDH) in a patient with absent corpus callosum.

## Case presentation

**Figure 1:**
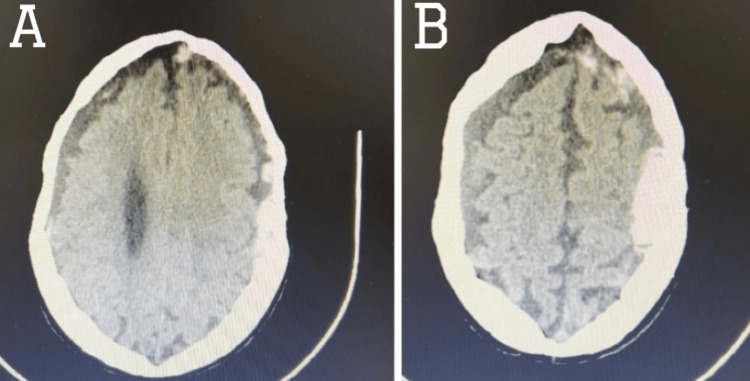
Axial cut of non-contrast-enhanced brain CT Axial cut of non-contrast CT scan of the brain showing bilateral subdural hematomas of different ages with (A) acute component on the left frontoparietal region with minimal mass effect and (B) associated minimal subarachnoid hemorrhage.

**Figure 2:**
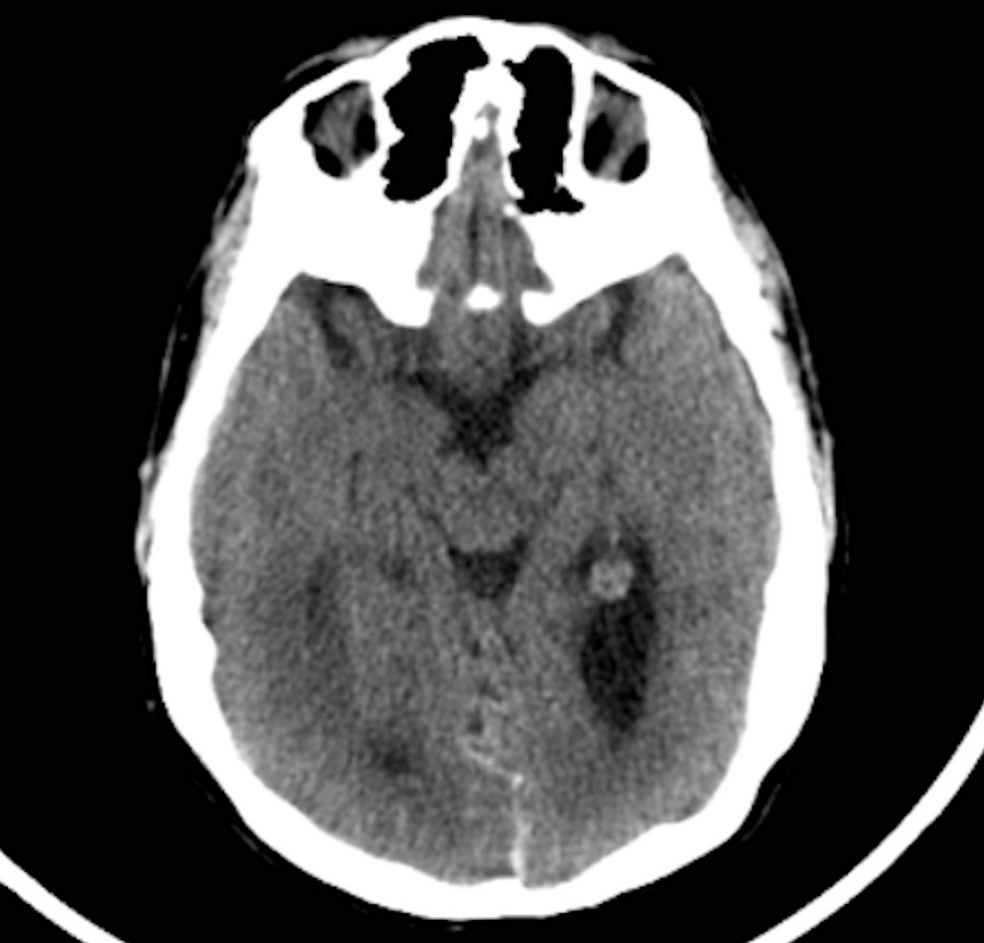
Non-contrast CT scan of the brain showing the absence of blood in the basal cisterns

A 39-year-old female who was a known case of epilepsy with corpus callosum agenesis, intellectual and social disability, and psychiatric issues on multiple medications presented to the emergency department with a headache and subtle right hemibody weakness in addition to a brief period of confusion. She was transferred to our hospital a few hours after being managed by a local hospital where a CT of the brain showed an acute on top of chronic subdural hematoma with convexity subarachnoid hemorrhage (Figures 1, 2). The patient and the family denied any history of direct head trauma or a clinical seizure.

**Figure 3:**
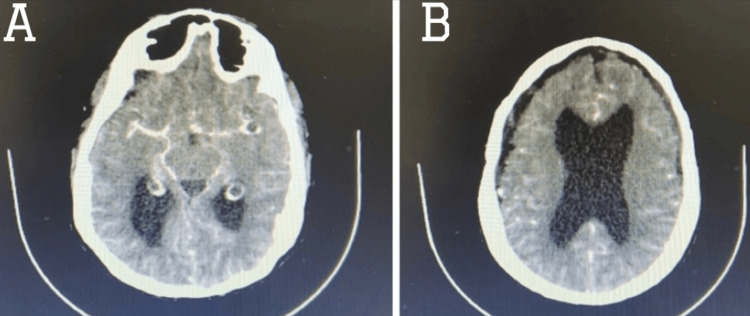
Axial cut of the CT angiogram of the brain Axial cut of the CT angiogram of the brain showed no vascular malformation in (A) the areas surrounding the circle of Willis and (B) the cortical frontoparietal areas.

**Figure 4:**
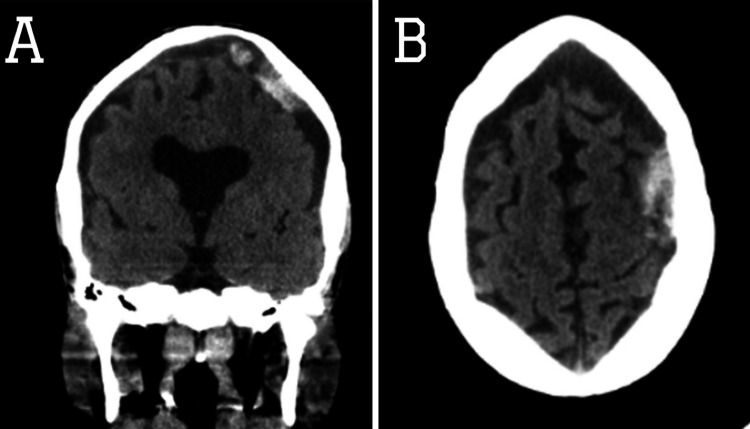
Non-contrast CT scan of the brain Non-contrast CT scan of the brain showing improvement in the size of the left frontoparietal subdural hematoma in both (A) coronal cut and (B) axial cut.

She was conscious and oriented, and according to the family, she was in her usual state of consciousness and alertness apart from the headache. On physical examination, her Glasgow Coma Scale (GCS) score was 14/15 E4 M5 V5 and there was no evidence of head trauma. Neuro examination including but not limited to muscle power, sensation, cranial nerves, and cerebellar examination was unremarkable. The patient was admitted to the intensive care unit (ICU) for continuous monitoring and conservative management for three days. Due to the absence of traumatic history, a CT angiogram of the brain was done to exclude any vascular lesions and it did not reveal any vascular malformation (Figure 3). Also, an MRI of the brain was done and did not show significant findings. The ICU course was uneventful and, after discussion with the family and the ICU team, it was decided to discharge the patient to the regular medical ward and then home after conservative management. Just upon discharge, on the third day following admission, the patient had dropped her level of consciousness and was reintubated and admitted back to the ICU. Her GCS dropped quickly to 8/15. An urgent non-contrast CT scan of the brain was obtained that only revealed improvement in the SDH thickness (Figure 4).

**Figure 5:**
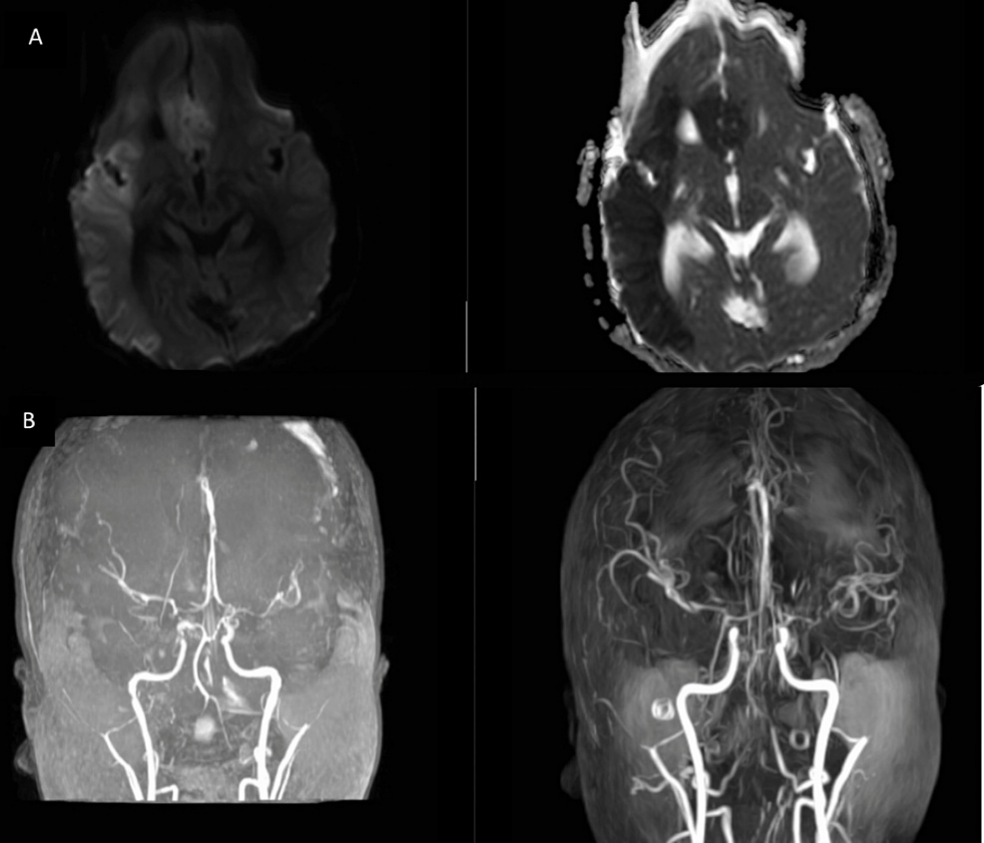
(A) Diffusion-weighted MRI of the brain showing bilateral brain ischemia. (B) Magnetic resonance angiography showing bilateral supraclinoid internal carotid artery and distal bilateral vasospasm

Conservative treatment was done, and antiepileptic drugs were started, with maintaining blood pressure and ruling out metabolic causes. She continued to deteriorate fast within hours to GCS of 5/15. The patient underwent an MRI with magnetic resonance angiogram (MRA), which showed severe global ischemia with patchy established infarction regions and severe bilateral vasospasm of the supraclinoid segment of the internal carotid arteries (ICA) and proximal large and distal small vessels (Figure 5).

Given the rapid deterioration, the patient was started on intravenous nimodipine, norepinephrine, and dexamethasone. With the consultation of our senior vascular neurosurgeon and the uncertain pathophysiology, an endovascular intervention was carried out under general anesthesia with the patient in the supine position. Initially, the systolic blood pressure was 120 mmHg, and the anesthetist was instructed kindly to maintain an average of 180 systolic till revascularization is established. Under ultrasonic guidance, a right femoral puncture was performed and a femoral sheath size 6 Fr was inserted into the right common femoral artery; the position was confirmed by fluoroscopy. The femoral sheath was connected to a heparinized arterial infusion line with heparin 4,000 units in 1 L of normal saline. Two catheters with the size of 6F and 5F were connected to a homeostatic valve attached to an infusion arterial pressurized line with heparin 4,000 units and 15 ml of nimodipine in 1 L of normal saline. The wire was followed by the catheters inserted through the femoral sheath and into the descending aorta until the aortic arch. An arch aortogram was performed. It was decided to start with the most severe side, i.e., the right side. The right common carotid artery origin was cannulated.

A cervical carotid angiogram was performed. The guiding catheter was advanced and parked at the distal cervical ICA segment. Selective carotid digital subtraction angiography (DSA) in anteroposterior (AP) and lateral view was performed. Severe cerebral vasospasm involving both proximal large and distal small cerebral vessels was found. A Scepter Balloon microcatheter (MC) with Traxcess 14 microguidewire (MGW) (MicroVention, Inc., Aliso Viejo, CA) was connected to a pressurized flush containing the same heparin and nimodipine. Technical difficulty with the angiography machine was encountered during this period. We continued the flush via the guiding catheter in the right ICA with nimodipine content. A mobile C-Arm with DSA capability was brought into the room for use. Finally, the angio machine worked again, and we continued the procedure. The Scepter Balloon MC was advanced and positioned at the ophthalmic segment of the right ICA. Verapamil 2 mg slow infusion was given to the patient.

The final DSA confirmed the excellent reversal of the vasospasm, with no complication. The guiding catheter was then repositioned and advanced and parked at the distal cervical ICA segment on the left side. Selective carotid DSA in AP and lateral view was performed. Severe cerebral vasospasm involving both proximal large and distal small cerebral vessels was also found. A Scepter Balloon MC with Traxcess 14 MGW connected to a pressurized flush containing the same heparin and nimodipine was advanced and positioned at the ophthalmic segment of the left ICA. The same previous technique for vasodilation was performed.

**Figure 6:**
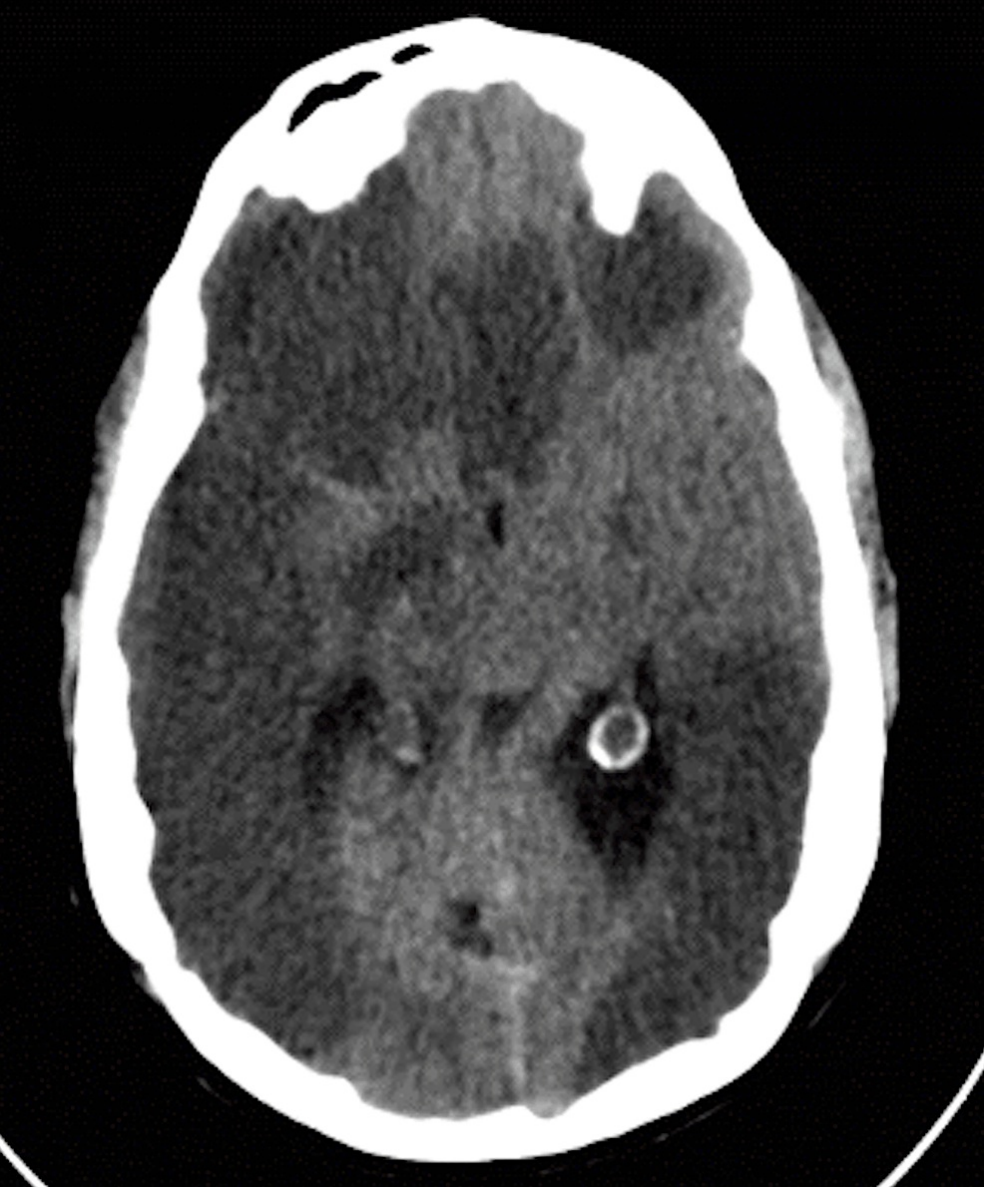
CT of the brain showing multiple hypodense regions representing established areas of brain infarction

The final DSA confirmed the excellent reversal of the vasospasm, with no complication. The anesthetist was informed to keep systolic blood pressure at 160-180 mmHg. All catheters were removed. The femoral sheath was removed and the closure device (Angioseal, Terumo Corporation, Somerset, NJ) used at the puncture site resulted in successful hemostasis. The patient was kept intubated and transferred to ICU for observation. Unfortunately, the patient did not improve and the CT scan showed multiple areas of infarction with edema and mass effect (Figure 6). The patient went into a deep coma for weeks without waking up and the family then decided to stop medical care. The patient died about a few weeks later in the hospital.

## Discussion

Cerebral vasospasm is a well-known clinical entity. Most of the described cases in the literature are reported following aneurysmal SAH. Other causes include but are not limited to traumatic brain injury, reversible vasoconstriction syndrome, post-brain tumor resection, and non-aneurysmal SAH [3]. There are also cases that were described following severe bacterial meningitis, vasculitis, sickle cell disease, and the use of sympathomimetic drugs such as cocaine [4]. Cerebral vasospasm following SAH is very well studied and generally occurs in 30% of patients with about 20% having a significant morbidity and/or mortality. Pathophysiology is poorly understood and probably involves irritation of the blood vessels by toxic products released into the arachnoid space [1,5,6]. Some genetic factors are recently implicated in the severity of clinical vasospasm [6,7]. Vasospasm usually occurs between three and 14 days after SAH with risk factors such as the severity of SAH, degree of motor deficit, as well as endovascular therapy being the most significant [5,8,9]. In traumatic brain injury cases, cerebral vasospasm typically occurs before the third day after trauma. Risk factors include severe head trauma, fever, presence of SAH, and young age [10-13]. In our case, the clinical vasospasm happened three days after spontaneous acute on top of chronic SDH with no major risk factors. It was very severe clinically leading to severe consequences. This was confirmed by imaging showing severe bilateral vasospasm in the supraclinoid segment of the ICA. Treatment of cerebral vasospasm is currently limited to supportive measures with nimodipine being the main medication to give especially in cases of aneurysmal SAH. Also, endovascular interventions such as intraarterial drug infusions as well as mechanical dilatations have proven effective in selected patients [3,11]. New treatments such as electrical stimulation (spinal cord stimulation, sphenopalatine ganglion stimulation) and cervical sympathectomy are undergoing trials of effectiveness [2,12]. Early recognition and detection of vasospasm are the cornerstones for a favorable outcome. The gold standard modality for the evaluation of vasospasm is cerebral angiography [13]. Transcranial Doppler sonography is widely used as a screening modality in the ICU. Although there are some technical issues attributed to its usage, in a recent study, the result of this modality was controversial for the detection of angiographically evident vasospasm in half of the patients. A promising result is shown with CT angiography as a screening test [13]. Moreover, the ability to detect vasospasm by MRA is highly correlated to that with DSA [11]. To the date of writing this report, this is the first case to report severe devastating vasospasm in a patient following acute spontaneous acute on top of chronic subdural hematoma. There is one case report of delayed cerebral vasospasm after surgical evacuation of a spontaneous subdural hematoma [14]. In their case, it was an acute hematoma in a young patient with significant mass effect who underwent a craniotomy and evacuation and developed cerebral vasospasm two days after the evacuation. They speculated that the surgery was the risk factor for developing vasospasm. The vasospasm was not severe and clinical improvement was achieved rapidly following medical management [14]. In our case, there were no such risk factors and the development and progression of the vasospasm were severe and unexpected. Our hypothesis is that the structural brain abnormality, namely, corpus callosum agenesis, diffuse brain atrophy, and unrecognized genetic abnormality, might have been a risk factor for developing such vascular reaction to this subdural hematoma [9]. The link between agenesis of the corpus callosum (ACC) and cerebral vasospasm is not that evident. ACC is one of the most common human anomalies occurring in 1.8 per 10,000 live births. This could be isolated or associated with other anomalies. It also can be part of a syndrome [9]. There are many syndromes associated with ACC described in the literature. There is no well-known association between ACC and cerebrovascular disease. Only a few reports have described the association between ACC, lipoma, and azygos anterior cerebral artery with an aneurysm [7]. In our case, there was no confirmed aneurysm on either computed tomography angiography or conventional angiography. Whether this fatal vasospasm was part of a hidden genetic syndrome or a coincidence is unknown to us.

## Conclusions

This case illustrates aggressive clinical vasospasm in a patient with spontaneous SDH with no history of trauma or brain aneurysm. Whether the underlying brain abnormality known in this patient was associated with an increased risk of developing cerebral vasospasm or is there a hidden genetic abnormality remains unknown. Further similar case reports and more observations are needed to help prevent such a complication in patients with corpus callosal agenesis. It is possible to see more cases of cerebral vasospasm associated with acute spontaneous SDH.
